# Fire history and treeline elevation in the Apennines: insights from pedo-anthracological analysis on Monte Cervati, Southern Italy

**DOI:** 10.3389/fpls.2025.1617687

**Published:** 2025-07-01

**Authors:** Giuliano Bonanomi, Adriano Stinca, Giandomenico Amoroso, Emilia Allevato, Giuseppina Iacomino, Gianluigi Mauriello, Riccardo Motti, Alfredo Nicastri, Francesca Bellucci, Mohamed Rida Abelouah, Luigi Di Costanzo, Mohamed Idbella

**Affiliations:** ^1^ Department of Agricultural Sciences, University of Naples Federico II, Portici, Italy; ^2^ Club Alpino Italiano, Presidente Comitato Scientifico Regionale and Sezione di Salerno, Salerno, Italy; ^3^ Department of Environmental, Biological and Pharmaceutical Sciences and Technologies, University of Campania Luigi Vanvitelli, Caserta, Italy; ^4^ Department of Biology, University of Naples “Federico II”, Naples, Italy; ^5^ Club Alpino Italiano, Presidente Gruppo Regionale and Sezione di Avellino, Avellino, Italy; ^6^ Laboratory of Aquatic Systems: Marine and Continental Environments (AQUAMAR), Faculty of Sciences, IbnZohr University, Agadir, Morocco; ^7^ AgroBioSciences (AgBS) Program, College of Agriculture and Environmental Sciences, Mohammed VI Polytechnic University, Ben Guerir, Morocco

**Keywords:** pedo-anthracology, charcoal, vegetation history, fire, black layer, SEM-EDS

## Abstract

**Introduction:**

The treeline elevation in the Apennines is significantly lower than its climatic potential, often attributed to historical anthropogenic disturbances such as fires, logging, and grazing. However, the specific impacts of individual disturbance events, particularly fires, on treeline dynamics remain unclear. This study investigates the relationship between treeline elevation and historical fire events using Monte Cervati (1,899 m a.s.l.) as a model system.

**Location:**

Monte Cervati, located in the Apennine Mountains, Italy.

**Methods:**

The current treeline elevation was mapped, and vegetation was characterized. Pedo-anthracological analyses were conducted in a sinkhole near the summit to reconstruct past vegetation and fire history. Charcoal samples from a paleosol layer were identified and dated to determine past fire events and vegetation composition.

**Results:**

The present treeline, composed exclusively of *Fagus sylvatica*, is situated at an average elevation of 1,710 m a.s.l., with higher elevations on northern slopes compared to southern ones. The vegetation above the treeline is dominated by small herbaceous species (*Plantago subulata*), with scattered shrubs such as *Daphne oleoides* and *Juniperus communis*. Notably, no *F. sylvatica* regeneration was observed above the treeline. Pedo-anthracological analysis revealed a charcoal-rich layer within a paleosol at 1,806 m a.s.l., dating back to approximately 4,800 BP. Charcoal analysis identified remains from herbaceous (*Dactylis*), shrubs (*Daphne*), and trees (*F. sylvatica*), indicating that past vegetation consisted of wooded grassland.

**Conclusions:**

Historical fire events likely played a crucial role in shaping the current treeline structure by eliminating the arboreal component and promoting the establishment of a predominantly herbaceous landscape. These findings suggest that fire disturbances have long-term effects on treeline dynamics, potentially contributing to the treeline depression observed in the Apennines today.

## 1 Introduction

The upper limit of the closed forest is a key landscape feature that characterizes most of the mountain ranges worldwide that reach sufficient elevation. The trees that reach the highest elevation are found in the Himalayas where *Juniperus tibetica* Kom. forest reaches 4,900 m a.s.l (Miehe et al., 2007). In the Alps, some conifers including the Swiss pine (*Pinus cembra* L.) and the larch (*Larix deciduas* Mill.), can reach 2200–2300 meters of elevation (Körner and Paulsen, 2004). The upper limit of the forest is undoubtedly the result of the complex interplay of temperature on the physiology of trees: the lower the temperature, the lower the elevation of the treeline. Ideally moving from the equator toward the poles, the elevation of the upper treeline progressively decreases from about 4,000 m a.s.l. in the tropics to sea level at the highest latitudes of Norway and Canada in the northern hemisphere and Tierra del Fuego in the southern hemisphere.

The upper treeline is commonly associated with decreasing air and soil temperature as elevation increases. Early studies claimed that minimum winter temperatures limited tree growth but subsequent studies have identified an isotherm of approximately 6.7° that characterizes the upper treeline on a global scale (Körner, 2012). This thermal limit is manifested on mountain slopes, as an imaginary boundary that trees cannot cross. When temperatures fall below this threshold, only prostrate shrubs, cushion plants and some herbs, mostly perennial scans complete their life cycle. The limit of ~6.7°C is not arbitrary but has deep eco-physiological foundations. A few decades ago, it was thought that low temperatures limited the process of chlorophyll photosynthesis, but later it was widely demonstrated that trees living at the treeline can efficiently perform this process down to temperatures close to 0°C (Körner, 1998), as long as the water in the soil remains unfrozen. In fact low temperatures, below ~6.7°C, strongly slow down cell division, distension and elongation (Körner, 2012).

While the eco-physiological factors like temperature and moisture availability (Sigdel et al., 2018; Bailey et al., 2021) determining the altitudinal position of the treeline are well understood on a global scale, various disturbances can significantly influence it at regional and local level, altering the maximum elevation at which forest formations are observed. Avalanches (Walsh et al., 1994), biotic interactions (Liang et al., 2016; Sigdel et al., 2024) in addition to grazing and logging are the factors that tend to lower, therefore to “depress” the treeline elevation relative to the potential climatic limit of a given species (Körner, 1998; Harsch et al., 2009). On the European continent, human activity over thousands of years has radically altered the vegetation in hilly and mountainous regions of the Alps, the Pyrenees, the Carpathians, and the Spanish mountain ranges (Colombaroli et al., 2010). This human activity has been particularly intense and pervasive on the Apennines (Bonanomi et al., 2020).

Stretching over 1,000 km along peninsular Italy, the Apennines form a mountain range where *Fagus sylvatica* is the dominant species at the upper limit of the forest, an environment that in a few cases is shared with the Bosnian pine (*Pinus heldreichii* Christ) on the Pollino massif and with the mountain pine (*Pinus mugo* Turra) on the Majella. *F. sylvatica* is an Eurasiatic deciduous species with the widest range among European trees, spanning from the Scandinavian Peninsula and the British Isles to Central and Mediterranean Europe. It is a tree that suffers heat and summer drought lacking specific fire-adaptive traits (Stinca et al., 2021). Therefore, in the Mediterranean basin it finds refuge in mountain areas, taking advantage of optimal climatic conditions such as cool and rainy summers. A study by our research group has quantified the maximum elevation reached by beech on 302 Apennine peaks covering a total of 3,622 km of treeline (Bonanomi et al., 2018). On average, beeches have their elevation limit at 1,589 m a.s.l., although there is considerable variability across different Apennine Mountain ranges. This study also reveals that in Pollino Park we find continental beeches that grow at the highest elevation in the world, reaching 2,140 m a.s.l. on the slopes of Monte Serra del Prete. Throughout the Apennines, the millenary action of human being, aimed at opening clearings in the forest to favor cultivation and grazing or to obtain timber or produce charcoal, has led to a drastic lowering of the maximum elevation reached by the forest (Bonanomi et al., 2020).

In the last decades, the study of fossil pollen gradually accumulated in the lakes and in the few Apennines peat bogs have allowed us a detailed reconstruction of the alternation of the main plant species during the glaciation and, more recently, in the Holocene. In particular, pollen analysis has provided valuable insights into the response of beech to the alternation of glaciations and to human activity at both continental and regional scales (Watts, 1985; Magri et al., 2006). However, only a few studies are available on pollen profiles at high elevation in the Apennines. For example, Magri (2007) demonstrated that, at Campo Felice, a site located at ~1500 m a.s.l., in the last 90,000 years the main tree taxa (e.g. *Abies*, *Fagus*, *Pinus*, *Quercus*) have alternated with expansions and subsequent contractions of population. The use of pollen as a paleo-vegetation proxy, however, also has its limitations. Pollen analysis depends on anoxic conditions for preservation, limiting its presence to environments like lakes, peat bogs, and wet sediments. Moreover, pollen is dispersed by the wind over long distances, often tens or even hundreds of kilometers. As a result, this method reflects vegetation changes on a regional or district scale but cannot provide detailed information on local events, such as shifts in the treeline. In contrast, detailed spatial information that cannot be obtained through pollen analysis can be gathered from the study of fragments of carbonized wood. Charred remains from the past periods could be preserved in the soil horizons and their interpretation allows us to reveal part of the history of the former vegetation with great spatial precision. Furthermore, charcoal being chemically inert is preserved in the soil for several millennia (Carcaillet and Thinon, 1996; Talon et al., 1998; Mercuri et al., 2015). Overall, soil charcoal analysis, or pedo-anthracology, combined with radiocarbon dating (^14^C) of charred wood fragments, is an effective method for reconstructing past forest dynamics with high spatial resolution (Thinon, 1978). It provides coupled localized insights into fire history and past vegetation composition, and, most importantly, can offer robust and detailed information on past altitudinal treeline shifts, shedding light on the changes of this critical ecological boundary (Carcaillet and Thinon, 1996). For example, distinct black layers in soil profile rich of charcoal residues have recently been successfully studied for vegetation reconstruction in New Zealand (McWethy et al., 2010). Unfortunately, in the Apennine the information available to date is limited and fragmentary and does not allow us to provide an overall picture of the changes that have occurred in the high-altitude forests (Benatti et al., 2019).

**Figure 1:**
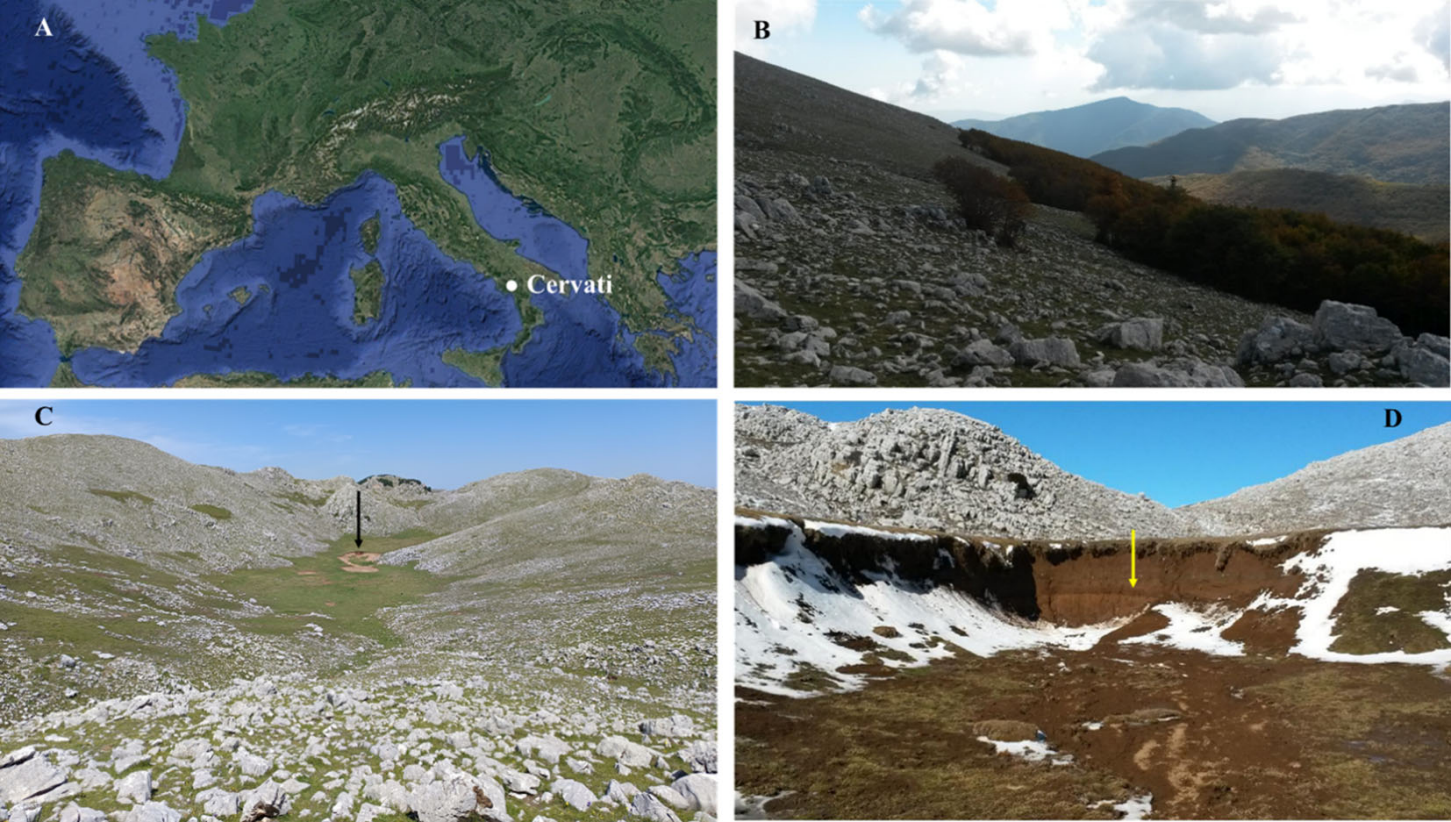
Location of Cervati massif in Southern Italy **(A)**; *Fagus sylvatica* treeline on the South slope at ~1,700 m a.s.l., with the transition between forest and the rocky grassland **(B)**, view of the summit sinkhole whose bottom is at an elevation of 1806 m a.s.l., (black arrow - **C**), and detail of the exposed profile of the sinkhole with the black layer located at about 150 cm depth (yellow arrow - **D**).

In this context, the aim of this study is to investigate the distribution of *Fagus sylvatica* on Mount Cervati, as a representative model of the Southern Apennines because of the carbonate substrate and the depressed treeline in term of elevation compared to the climatic potential. The study first quantified the distribution of the current treeline as well as its current dynamics of regeneration and recolonization of the summit areas above the treeline. In addition, we studied a distinct black layer present in a sinkhole located close to the mountain peak (**Figure 1**). We hypothesized that such a black layer represents the traces of ancient fires that would transform the vegetation of the summit of Cervati. Consequently, we used the pedo-anthracological approach based on optical and scanning electron microscopy and energy dispersive spectroscopy (SEM–EDS) for taxonomic identification of the charcoal samples and to evaluate the long-term alterations of wood buried in the soil. Radiocarbon dating of charcoal samples was conducted to determine the period in which historical fires occurred. The specific hypotheses tested were as follows:

- The *F. sylvatica* treeline is depressed in terms of elevation compared to the species’ potential.
- *F. sylvatica* is unable to recolonize open areas above the treeline.
- Past historical fires may have contributed to depressing the upper limit of the treeline.

## 2 Materials and methods

### 2.1 Study sites description

With 261 peaks rising above 2,000 m a.s.l., the 1,200 km long Apennine mountain range spans 38°N to 44°N. *Pinus nigra* J. F. Arnold subsp. *nigra* and *Pinus heldreichii* Christ subsp. *Leucodermis* (Antoine) E. Murray are rare and remnant plant populations are found in the middle and southern regions of the Apennines, respectively, while *F. sylvatica* dominates the treeline along the chain (Bonanomi et al., 2018). Limestone makes up most of the substrate, with sporadic arenaceous-peliticflysch in the Laga groups (https://www.pcn.minambiente.it/GN). With mean temperatures between 0 and 11°C in January and 24 and 28°C in July, the climate is a mountainous variation of the Mediterranean type. Total annual precipitation varies between 800 and 3,000 mm, with regular winter snowfall occurrences above 1,000 m a.s.l.

The present study was carried in the Mt. Cervati located in the National Park of Cilento, Vallo di Diano and Alburni, southern Italy (40°17’8.97”N, 15°29’11.18”E; 1,899 m a.s.l.). Study site also fall within the Site of Community Importance IT8050024 “Monte Cervati, Centaurino e Montagne di Laurino”, and Special Area of Conservation IT8050046 “Monte Cervati e dintorni”. The massif is composed of a mainly carbonates substrate with intense karst activity, ensuring a year-round water supply to neighboring territories. At lower elevations (0–800 m a.s.l.) annual precipitation averages are around 800 mm, while at higher elevations rainfall exceeds 1000 mm. The natural vegetation of the area is characterized by different forest coenosis that cover all slopes of the massif, while in the summit area there are rocky meadows often used for grazing. The forests, especially at lower elevations, are often coppice and composed of many broadleaf trees such as *Castanea sativa* Mill., *Quercus cerris* L., *Quercus pubescens* Willd. subsp. *pubescens*, *Acer opalus* Mill. subsp. *obtusatum* (Waldst. & Kit. ex Willd.) Gams, *Ulmus minor* Mill. subsp. *minor*, *Ostrya carpinifolia* Scop., etc. Beech forests appear from about 900 m a.s.l. and, in addition to *F. sylvatica*, only rarely show other tree species (e.g. *Taxus baccata* L., *Ilex aquifolium* L., etc.). This vegetation type can be attributed to the habitat “Apennine beech forests with *Taxus* and *Ilex*” (code 9210*, Habitats Directive 92/43/EEC), widespread in the Italian Peninsula and Sicily in the supratemperate bioclimatic plan and rarely in the upper mesotemperate (Zitti et al., 2016). In the plant communities of the summit area of the massif, perennial herbaceous species are very abundant, many of which are endemic and rare (e.g. *Potentilla rigoana* Th. Wolf, *Cynoglossum magellense* Ten., *Pedicularis elegans* Ten., *Gentianella columnae* (Ten.) Holub, *Saxifraga exarata*Vill. subsp. *ampullacea* (Ten.) D.A. Webb (Santangelo et al., 1994). (**Figure 1**). Also noteworthy are the shrublands with *Lavandula austroapennina* N.G. Passal., Tundis & Upson which can be observed on some rocky slopes of the massif. This species is a recently described endemic of the southern Apennines (Gravina et al., 2023).

### 2.2 Current treeline assessment

Treeline assessment was conducted for Mt. Cervati following the approach of Bonanomi et al. (2018), that recorded treeline elevation of all peaks with an elevation above 1,500 m a.s.l. across the Apennines. In this study treeline elevation was carefully mapped using the tool Google Earth Pro™ (Google, Inc. Mountain View, CA, USA) using images spanning from 2004 to 2018. The treeline elevation was recorded by manually delineating the boundary between forest and grassland on the four different aspects (north, east, south and west) of mountain. Afterward, the digitized drawn lines were carefully measured for their maximum, average, and minimum elevations above sea level. The treeline elevation measured with satellite images was compared with ground measurements using GPS (Garmin Montana 750i) at forty points in total, ten for each exposure.

### 2.3 Current vegetation beyond the treeline and *Fagus sylvatica* regeneration

The current natural vegetation of the summit study site was analyzed through field surveys conducted in June 2024, followed by detailed taxonomic analyses in the laboratory. Sampling was performed in eight 1 × 1 m plots (i.e. 4 on the sinkhole and 4 on the surrounding slopes) recording all vascular taxa and visually estimating their absolute percentage cover. The taxonomic identification was carried out using Pignatti et al. (2017a, b, 2018, 2019), while nomenclature and taxa delimitation followed the checklist of Italian vascular flora (Bartolucci et al., 2024). The collected plant material was stored in the Herbarium Austroitalicum (IT).

The surveys concerning *F. sylvatica* regeneration was conducted at the treeline during the summer of 2024. Four rectangular transects (10 × 200 m), or belt transects, was established for each aspect for a total of sixteen transects. The transects were spaced at least 200 meters from the treeline to open grassland. First, we measured the overall height of *F. sylvatica* at the treeline using a telescopic graduation meter. In each transect, all beech seedlings and samplings were counted and the distance from the treeline was measured. In addition, the total cover (%) of shrubs, herbaceous species and outcrops was quantified in each transect.

### 2.4 Soil sampling in the sinkhole, anthracological analysis and radiocarbon dating

**Figure 2:**
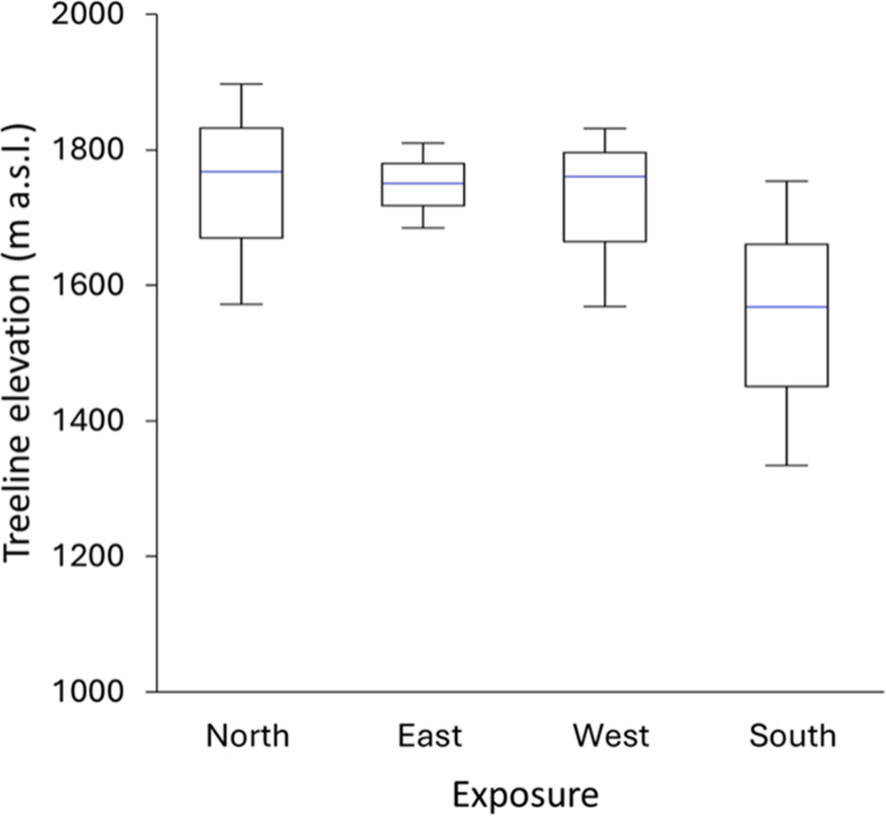
Elevation of *F. sylvatica* treeline according to exposition; each box represents the average value; upper and lower whiskers reach are minimum and maximum values observed.

In June 2022, sampling activities were conducted in the summit sinkhole area, located at an elevation of 1,816 m a.s.l. (40°17’8.97”N, 15°29’11.18”E), just below the mountain peak (**Figure 1**). To investigate Holocene vegetation and fire history, we applied a soil charcoal analysis approach (Carcaillet and Thinon, 1996; Robin and Nelle, 2014). Soil samples were collected along an exposed profile of the sinkhole at four depths: 50 cm, 100 cm, 150 cm at the black layer suspected of being rich in charcoal residue due to its dark color, and near the bottom at 200 cm depth. Approximately 10 kg of soil was collected for each depth (**Figure 2**). The soil samples were placed in polyethylene bags and transported to the laboratories of the Department of Agriculture, where it was air-dried and then stored at room temperature.

The soil was subsequently washed with water over a column of sieves with calibrated mesh sizes of 4 mm, 2 mm, 1 mm, and 0.5 mm to remove fine sediment. Charcoal fragments were extracted under a binocular microscope and stored for further analysis. Identification of the charred wood fragments was performed by examining the three anatomical planes of the wood under a reflected light microscope, with magnifications ranging from 50x to 1000x. The identification was based on a comparison with wood anatomy atlases (Greguss, 1959; Schweingruber, 1990) and electronic identification keys (Heiss, 2002; Dallwitz et al., 1995; InsideWood, 2004-present). The Anthracological approach is often criticized because wind can carry charcoal particles from fires occurring at lower altitudes up to the treeline and beyond. However, experimental studies and trap-based observations summarized in Whitlock and Larsen (2001) have shown that macroscopic charcoal (>125–250 µm) is not transported far from the fire margin and typically deposited within a few meters from the fire source.

### 2.5 Radiocarbon dating

Three charcoal fragments previously identified as *Dactylis* were selected for radiocarbon dating at the laboratories of the Department of Mathematics and Physics, University of Campania “Luigi Vanvitelli.” We selected only charcoal fragments from herbaceous species to minimize potential discrepancies between the plant’s lifespan and the fire event, thereby avoiding the well-known “old wood” effect—where dating the inner part of a large, long-lived tree reflects the year that specific part of the wood was formed, rather than the year the tree actually burned (Geib, 2008).

Conventional ^14^C ages were calibrated using the OxCal 4.4 [v. 175] online program (Ramsey, 2009) and the IntCal20 calibration curve data (Reimer et al., 2020). To better constrain the burning event(s), the ^14^C dates were tentatively pooled into different groupings using the R_Combine function of OxCal, which statistically merges dates assumed to derive from the same event and applies a χ² test to evaluate their internal consistency, in order to verify the assumption that the burning corresponds to a single date or to multiple distinct events (Ramsey, 2009).

### 2.6 Charcoal scanning electron microscopy and energy dispersive spectroscopy

We employed scanning electron microscopy and energy dispersive spectroscopy (SEM–EDS) to analyze *Fagus sylvatica* wood and *Dactylis* spp. herbs. Samples, approximately 1 × 0.5 × 0.3 cm in dimension, were attached to SEM stubs using conductive carbon paint with double-sided adhesive tape. To achieve topographical mapping of the chemical elements within these samples, SEM imaging was conducted in tandem with energy-dispersive X-ray spectroscopy (EDS). EDS utilizes a focused beam of accelerated electrons to induce fluorescence emissions from the elements present in the samples. The energy of these emitted X-rays is unique to each element, enabling identification and analysis of the chemical composition within the outermost layers (spanning a depth of several hundred nanometers to approximately 2 μm) of the *Fagus sylvatica* wood and *Dactylis* spp. herbs. A field emission scanning electron microscope (SEM, FEI Nova NanoSEM 450) installed at the Centro di Microscopia within the Dipartimento di Scienze Chimiche, University of Napoli Federico II, was used to study the samples’ morphology. The images were acquired by collecting secondary electrons (SE) with a Bruker ETD detector, all operating at room temperature. The imaging parameters included a working distance of 4.9 mm and an acceleration voltage of 3.00 kV. Analysis of the SEM images was performed using the AZtecCrystal software package, which facilitates the processing of data obtained via electron backscatter diffraction (EBSD) (Wilkinson and Britton, 2012).

## 3 Results

### 3.1 Current treeline elevation

Overall, current mean treeline elevation in Cervati was 1,710 m a.s.l., although considerable variability among exposure was found with the highest values in northern and lowest in southern expositions (1,743 and 1,554 m a.s.l., respectively) (**Figure 2**). The absolute maximum treeline elevation was 1,891 m a.s.l., just few meter below the top of the mountain (i.e. 1,899 m a.s.l.), with the lowest values of only 1,342 m a.s.l. on the southern exposition. Finally, we found a notable Δ maximum – minim treeline elevation of 276 m, with a highest Δ for southern exposition (409 m) compared to eastern (122 m), western (256 m) and norther (317 m) expositions.

### 3.2 Current vegetation beyond the treeline and *Fagus sylvatica* regeneration

Our field surveys carried out above the treeline have highlighted the current presence of two vegetation types related to substrate conditions (**Table 1**). The periodically flooded and almost flat soil of the sinkhole shows a community with a low number of vascular species (average species richness per m^2^ of 5.25 ± 1.23 standard deviation) and dominated by *Plantago subulata* (average cover 58.75%). Instead, the rocky slopes surrounding the sinkhole are characterized by more biodiverse cenoses (average species richness per m^2^ of 9.25 ± 1.98 standard deviation) with a prevalence of *Globularia cordifolia* L. subsp. *Bellidifolia* (Nyman) Wettst. (average cover 41.25%). Both vegetation types are heliophilous, composed mostly of hemicryptophytes, and species of conservation interest (i.e. Italian endemic species such as *Achillea tenorei* Grande, *Potentilla calabra* Ten., and *Potentilla rigoana* Th. Wolf), and intensively grazed by horses and cattle.

**Table 1:** Vegetation report of sinkhole and slope plant communities from Mt. Cervati.

Plot code	Sinkhole 1	Sinkhole 2	Sinkhole 3	Sinkhole 4	Slopes 1	Slopes 2	Slopes 3	Slopes 4
Elevation (m a.s.l.)	1818	1818	1818	1818	1841	1854	1829	1822
Exposure	SSE	ESE	–	SE	NNE	NNE	SW	SSW
Slope (°)	1	1	–	1	60	50	50	40
Shrub layer height (m)	–	–	–	–	–	–	–	–
Herbaceous layer height (m)	0.02	0.02	0.02	0.01	0.2	0.3	0.3	0.3
Total vegetation cover (%)	70	95	90	40	70	70	60	70
Shrub layer cover (%)	–	–	–	–	–	–	–	–
Herbaceous layer cover (%)	70	95	90	40	70	70	70	60
Bare soil cover (%)	3	0	1	5	1	0	2	2
Rocks and stones cover (%)	2	0	0	0	24	28	25	35
Litter cover (%)	25	5	9	55	5	2	3	3
Taxon name	Vegetation composition in term of taxon cover (%)							
*Achillea tenorei* Grande	2							
*Anthyllis montana* L. subsp. *jacquinii* (Rchb.f.) Rohlena					10		1	0.5
*Anthyllis vulneraria* L. s.l.						0.5		
*Campanula scheuchzeri* Vill. s.l.					1			
*Carex kitaibeliana* Degen ex Bech.					2	10	10	5
*Draba verna* L. subsp. *verna*	0.5							
*Edraianthus graminifolius* (L.) A.DC. ex Meisn. subsp. *graminifolius*					0.5	0.5		
*Festuca* sp.							1	0.5
*Galium lucidum* All.						0.1	0.1	0.1
*Globularia cordifolia* L. subsp. *bellidifolia* (Nyman) Wettst.					50	60	30	25
*Helianthemum nummularium* (L.) Mill. s.l.					1	1	10	15
*Hippocrepis comosa* L. subsp. *comosa*							1	
*Onobrychis alba* (Waldst. & Kit.) Desv. subsp. *pentelica* (Hausskn.) Nyman						0.1	0.1	1
*Pimpinella tragium* Vill.					1		1	1
*Plantago subulata* L.	65	80	60	30				
*Poa alpina* L.		15	25	5				
*Potentilla calabra* Ten.	2	1						
*Potentilla rigoana* Th.Wolf								0.1
*Sagina glabra* (Willd.) Fenzl	0.5	1	3					
*Scleranthus polycarpos* L.	3	2	0.2	10				
*Sempervivum tectorum* L.					0.5			
*Thymus* sp.					20	0.1	20	15
*Trifolium repens* L.	0.5	0.5	5					

Above treeline, shrub cover was very low (<5%) with scattered *Juniperus communis* L. And *Daphne oleoides* Schreb. subsp. *oleoides*. Concerning *F. sylvatica* regeneration, seedlings were completely absent above the treeline irrespectively of elevation and exposure of the transect. In fact, despite intensive searches in the transects arranged on the four sides of the mountain, no *F. sylvatica* seedlings or saplings were observed beyond the treeline.

### 3.3 Charcoal dating and identification

**Figure 3:**
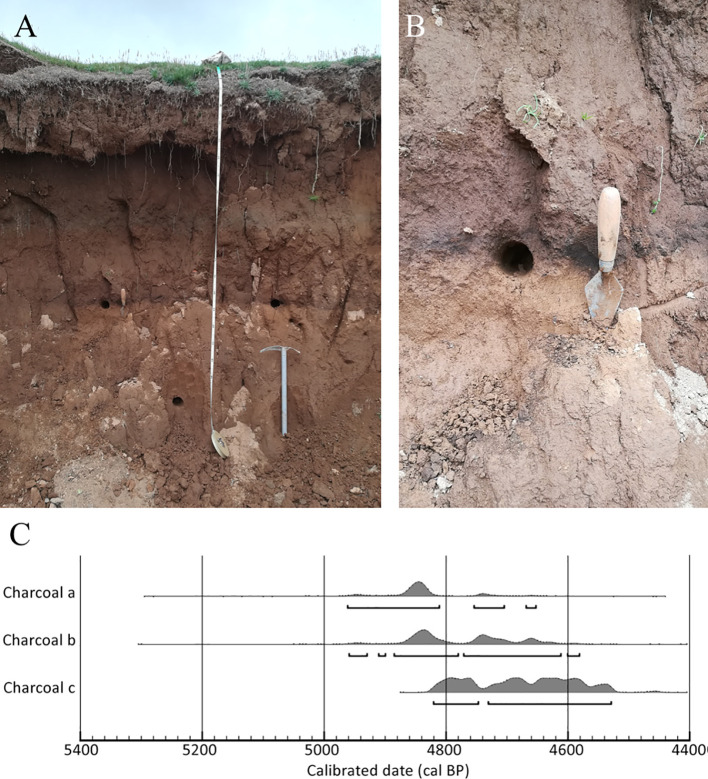
Soil profile in the sink hole with the black layer at ~150 cm depth, note the ice axe for scale **(A)**, detail of sampling point **(B)**, and multi-plot of calibrated date of the three charcoal samples **(C)**.

**Figure 4:**
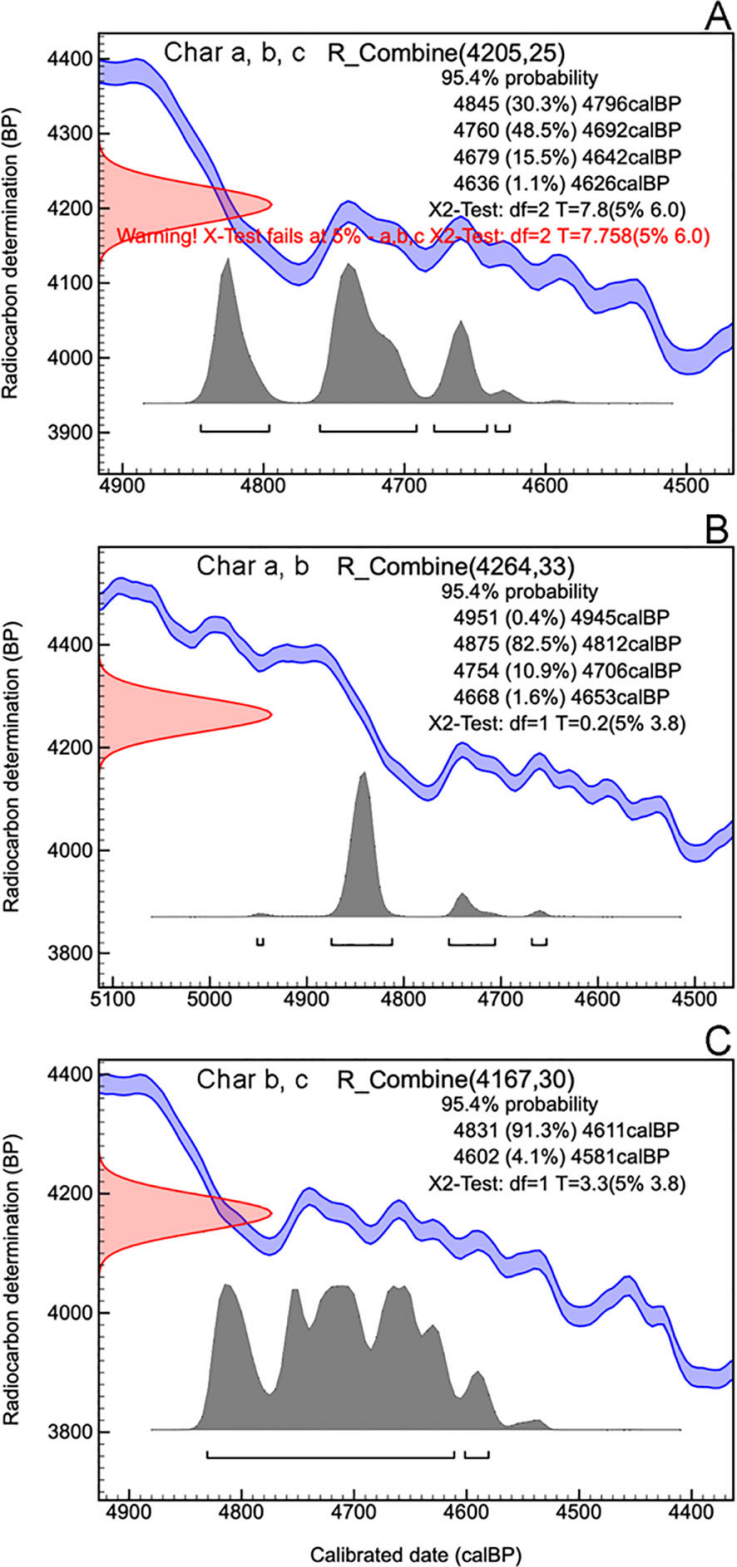
OxCal plot of the combined date of samples Charcoal a, Charcoal b and Charcoal c **(A)**, of the combined date of charcoal samples Charcoal a and Charcoal b **(B)** and of the combined date of charcoal samples Charcoal b and Charcoal c **(C)**.

Concerning charcoal dating, the three single-fragment charcoal samples from the black layer, gave calendar year dates, after calibration at 2σ intervals, ranging from 4845 to 4529 cal BP (**Table 2**, **Figure 3**). The results obtained for all dated samples could not be combined, as the χ² test failed [χ² test: df = 2, p = 7.8 (5%: 6.0)]. However, when sample b was combined individually with sample a and then with sample c, good statistical significance was achieved [respectively: χ² test, df = 1, p = 0.2 (5%: 8.8) and χ² test, df = 1, p = 3.3 (5%: 3.8)]. This resulted in two combined date ranges: 4951 to 4653 (median = 4841) and 4831 to 4581 (median = 4710), both with a 95.4% probability (**Figure 4**).

**Table 2:** Radiocarbon dating results of the three charcoal samples retrieved from the soil profile close the summit of Mt. Cervati.

Sample name	^14^C Age BP	Median	Calibrated age (Cal BP, 2σ)		
Charcoal 1	4274 ± 41	4845	4962	–	4653
Charcoal 2	4246 ± 53	4790	4959	–	4581
Charcoal 3	4130 ± 36	4674	4821	–	4529

The table presents the conventional radiocarbon ages (expressed in years BP, Before Present = 1950), the associated standard deviations, and the calibrated age ranges at 2σ (95.4% probability), providing the most probable calendar age intervals for each sample. The “Median” column indicates the median calibrated age based on the calibration curve.

No charcoal was detected at depths of 50 and 100 cm. In contrast, at a depth of 150 cm, a total of 76 charcoal fragments were recovered over the sieve 0.5 mm. Three taxa were identified: the tree *Fagus sylvatica* (n = 2), the shrub *Daphne* sp. (n = 6), and a perennial grass of the Poaceae family, *Dactylis* sp. (n = 25); 43 charcoal fragments remained unidentified. A few very (n= 10) small and poorly preserved charcoal fragments were found at the bottommost level (200 cm), but they could not be identified due to their extremely poor state of preservation coupled with their small size.

**Figure 5:**
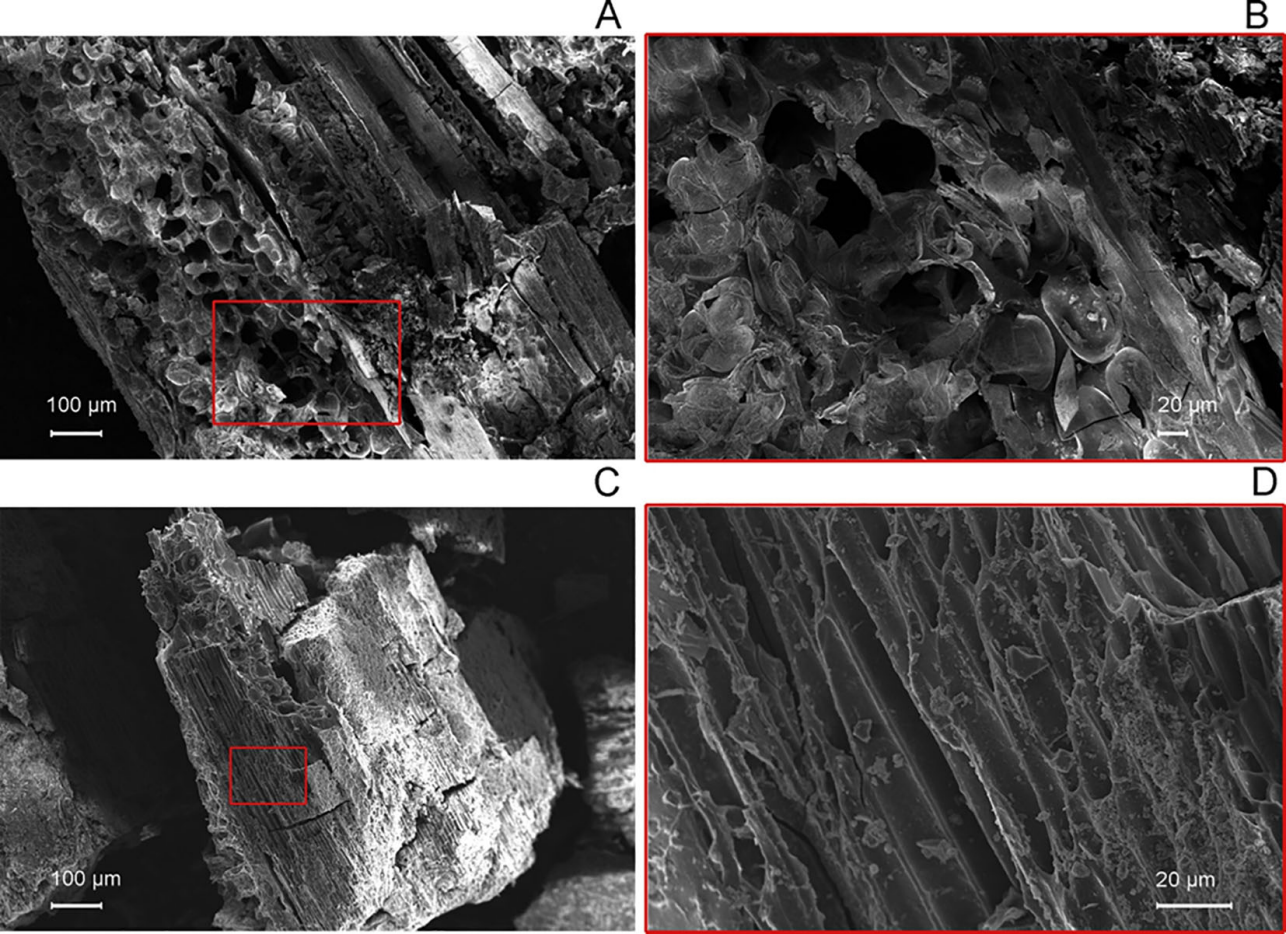
Scanning electron microscope images of the anatomical structure in the longitudinal tangential plane of *Fagus sylvatica* wood, showing ray parenchyma cells in a multiseriate ray **(A, B)**, and of vascular tissues in the longitudinal plane of *Dactylis* spp. herbaceous samples **(C, D)** at different magnifications. The images on the right provide a magnified view of the red-squared area from the corresponding left image.

Scanning electron microscopy (SEM) revealed distinct anatomical features and taphonomic alterations in the ancient *Fagus* and *Dactylis* samples, as shown in **Figure 5**. *Fagus* (**Figures 5A, B**) displayed the expected thick-walled cells arranged in a regular pattern (**Figure 5A**), typical of hardwood trees, but with evidence of cell wall degradation (**Figure 5A**), consistent with an ancient sample. In contrast, *Dactylis* (**Figures 5C, D**) exhibited thinner cell walls and a more varied cellular arrangement (**Figure 5C**), characteristic of grasses. Notably, the *Dactylis* sample showed adhered depositions on cell surfaces (**Figure 5D**), giving it a rock-like solidified appearance, indicative of mineralization. The preservation of key cellular details in both samples, despite these alterations, highlights the resilience of plant tissues and the power of SEM analysis for investigating ancient plant remains.

**Figure 6:**
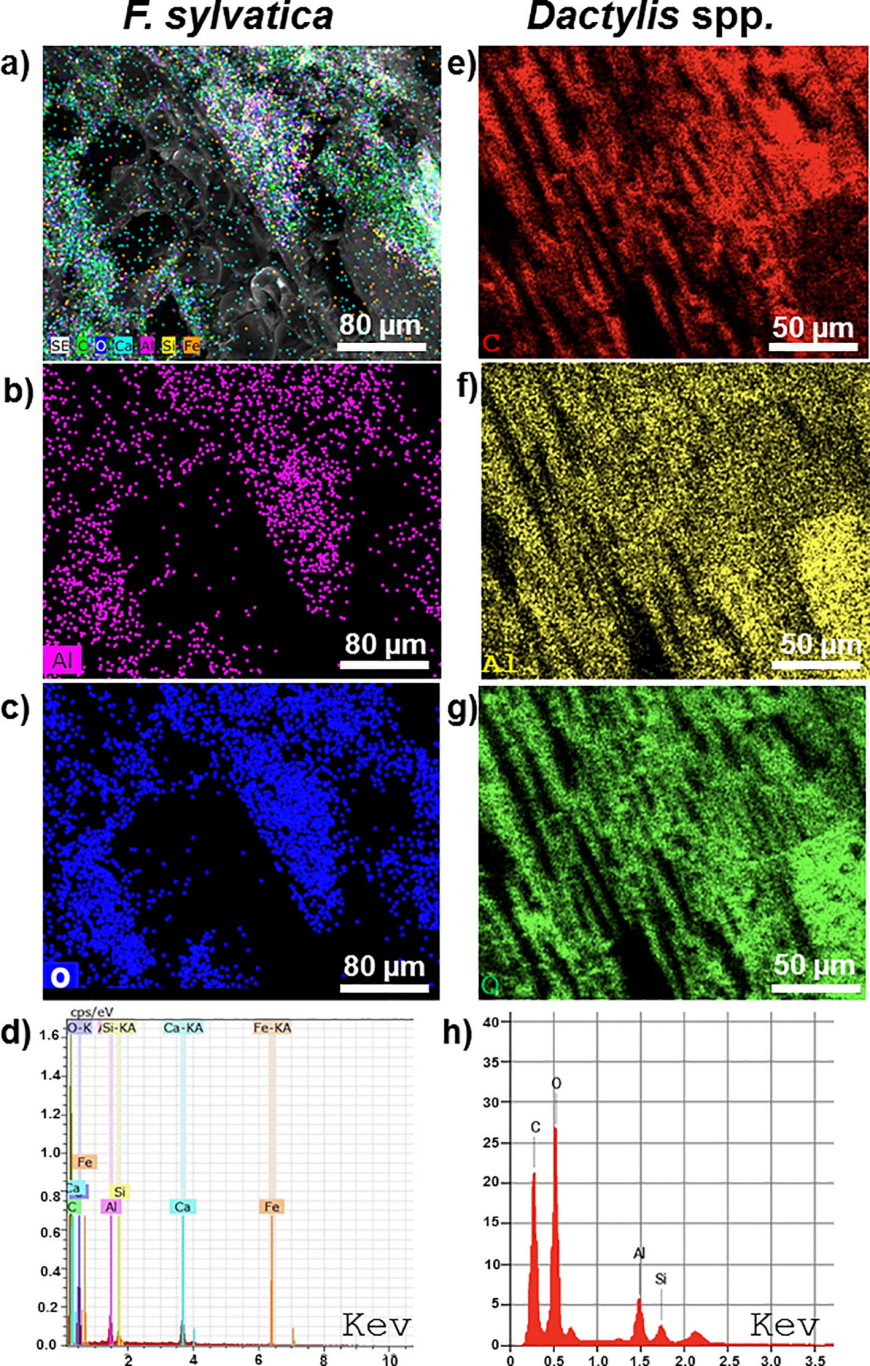
Scanning electron microscope (SEM) images combined with Energy-Dispersive X-ray Spectroscopy (EDS) mapping and X-ray spectra emission showing the elemental composition of *F sylvatica* wood (left) and *Dactylis* spp. herbs (right). **(a)** Surfaces of *F sylvatica* charcoal, the elemental maps illustrate the accumulation of Al_2_O_3_. **(b, f)** Localized accumulation of aluminium (Al) on the surfaces of *F sylvatica* wood **(b)** and *Dactylis* spp. herbs **(f)**. **(c, g)** EDS elemental mapping showing the distribution of oxygen (O) on the surfaces of *F. sylvatica* wood **(c)** and *Dactylis* spp. herbs **(g)**. **(d, h)** EDS X-ray emission spectra from representative areas on *F sylvatica* wood **(d)** and Dactylis spp. herbs **(h)**. **(e)** SEM image showing the carbon (C) distribution on a closer view of the surface of *Dactylis* spp. herbs. The localized accumulation of aluminium on both types of charcoal corroborates the radiodating data, as this process is particularly slow and occurs in paleosols that have undergone calcium leaching, which has not been found in large quantities as expected in recent soils of carbonate matrix. Sample scales are indicated in the respective images.

SEM-EDS analysis provided crucial evidence linking the ancient *Fagus* and *Dactylis* samples to the Al_2_O_3_-rich suggesting that the charcoal is buried in a paleosoil (Sheldon and Tabor, 2009). As expected, the EDS mapping clearly showed a layer of Al_2_O_3_ covering both samples, consistent with the characteristic accumulation of this mineral in paleosols. The EDS maps and X-ray spectrum further confirmed the presence of aluminum (Al) in both the *Fagus* and *Dactylis* samples, as shown in **Figure 6**. Notably, a mapping consistent with uniform distribution of Fe_2_O_3_ was detected in *Fagus* but not in *Dactylis*, possibly indicating iron-related mineral formation within the degrading wood tissues. While both samples showed some silicate presence, the levels were higher in *Dactylis*, consistent with the expected silica accumulation in grasses, contributing to its rock-like appearance. This combined SEM-EDS analysis not only confirmed the presence of Al_2_O_3_, a hallmark of the paleosol environment, but also revealed how this mineral and other elements interacted with the plant tissues, influencing their preservation and taphonomic alteration.

## 4 Discussion

### 4.1 Current treeline elevation and *Fagus sylvatica* regeneration

The treeline of Mt. Cervati is, on average, at 1,710 m a.s.l., which is higher than the average of the Apennine chain, which is 1,589 m a.s.l (Bonanomi et al., 2018). However, this value is decidedly lower than the estimated ecological potential of *F. sylvatica* at these latitudes, which is higher than 2,000 m a.s.l (Bonanomi et al., 2018). In fact, treelines of *F. sylvatica* higher than 2000 m a.s.l. have been described in the Apennines both further south, such as in Pollino (Rita et al., 2021), and further north, such as on Mount Argatone, Mount Greco and on Majella (Bonanomi et al., 2020). This implies that the Cervati treeline is depressed, on average and with respect to its climatic potential, by at least 300 m. The in-depth analysis by slope has also revealed that on the southern slope the treeline is on average 187 m lower than on the other slopes, where the minimum values are also found at only 1,342 m a.s.l., much lower than the absolute maximums located on some valleys on the northern slope that almost reach the top of the mountain at 1,891 m a.s.l. Our findings that the treeline elevation was consistently lower on warmer, south-facing slopes, is consistent with earlier research across the Apennines (Bonanomi et al., 2020). This pattern, though the underlying mechanisms remain unclear, may result from a synergistic interplay between human disturbance (such as logging and grazing pressure) and climatic restrictions (such as summer dryness), which could result in the loss of *F. sylvatica* canopy viability and regeneration capability.

When compared to the predicted climatic potential, the depression of the treeline elevation at Mont Cervati suggests a widespread anthropogenic influence. Our hypothesis states that within mountain groups, we found the coexistence of a highly depressed treeline, in several cases with elevation of only ~1,300 m a.s.l., alongside a very high treeline, reaching up to nearly 1,900 m a.s.l. The co-occurrences of such variability in treeline elevation across sites that are a few kilometers apart suggest that factors other than climate control this pattern. Given this, we propose that high elevation treeline exists in remote, inaccessible and steep valleys which have historically been shielded from anthropogenic disturbances, both past and present. Conversely, very depressed treeline is located in accessible areas that have been heavily exploited in previous centuries.

Despite intensive research, no seedling or sapling regeneration was detected above the treeline regardless of elevation and exposure. This observation suggests that the open grassland environment poses significant challenges to *F. sylvatica* to recolonization. According to Allegrezza et al. (2016), even at the treeline plants outside of the canopy cover on south-facing mountain slopes may face extremely high soil and air temperatures (up to 38°C) and severe summer droughts. These conditions significantly limit the ability of *F. sylvatica* to regenerate on open patches. In this approach, *F. sylvatica* may act as an ecosystem engineer by modifying forest microclimate through its canopy cover, thereby facilitating seedling establishment. As a result, we hypothesize that the treeline depression on southern mountain faces is a consequence of *F. sylvatica* reduced engineering potential following canopy removal. When left intact, this species exerts positive feedback on microclimate, reducing wind effects, buffering temperature extremes, and enhancing soil and air moisture (Hastings et al., 2007). A recent study (Bonanomi et al., 2021) demonstrated that in the presence of shrubs such as *Juniperus communis* and *Pinus mugo*, *F. sylvatica* can rapidly recolonize areas beyond the treeline. This is due to the facilitative effects of the microclimate modulated by the crowns of the nurse shrub, thereby improving the conditions for *F. sylvatica* establishment. We hypothesize that the buffering effect of plant canopies will be more significant in southern than in northern aspects, where air and soil temperature are already buffered by the lower solar radiation, because self-shading cushions local microclimate (Rita et al., 2021). The limited ability to recolonize open areas above the treeline has been reported in several areas of the globe and the combination of summer drought with the shortness of the growing season are formidable constraints for many species living at the upper treeline (Elliott et al., 2020; Lyu et al., 2019; Bar-On et al., 2022). Such factors undoubtedly limit the ability of the treeline to advance even where thermal limitations are less severe due to global warming (Harsch et al., 2009).

### 4.2 Past fire causes depression of current treeline?

European mountain ranges including the Alps, the Pyrenees and the Apennines have a history of intense disturbances such as recurrent fires, forest clearing, and intensive grazing that have profoundly altered vegetation across various elevation (Gobet et al., 2003; Bal et al., 2011). Human activity has been particularly intense along the Apennines as demonstrated by the pervasive presence of kiln platforms, observed from low elevation up to the treeline at approximately 2,000 m a.s.l (Carrari, 2015; Bonanomi et al., 2020). Studies based on pollen profiles, such as those conducted at Lake Monticchio located 73 km from Monte Cervati, have reconstructed vegetation fluctuations over the last 76,300 years (Watts et al., 1996), but with low resolution and therefore at a regional scale. Studies based on anthracological remains found in archaeological sites are more detailed. Near the study area, the Serratura cave (Di Pasquale et al., 2020) and the Maria Colombo cave (Belli, 2016) were studied. In both cases, the studies enable the reconstruction of the vegetation surrounding the cave area, although it cannot be ruled out a selection effect by populations that collected wood for cooking and heating. Furthermore, both caves are present at relatively low elevation, 830 m for Maria Colombo (Belli, 2016) and at sea level for the Serratura cave (Di Pasquale et al., 2020), therefore with limited relevance as regards the vegetation dynamics at the upper limit of the forest. In this context, the discovery of the black layer in the summit sinkhole of Monte Cervati provides a unique opportunity to reconstruct, with temporal precision, the changes in vegetation regarding the peaks of the Apennine mountains.

The localized accumulation of numerous charcoals remain within a single soil layer demonstrates that the summit of Monte Cervati experienced significant fire event(s). To estimate a probable calendar age for the burning event, we tentatively combined all ^14C dates using the OxCal function R_Combine. However, the χ² test indicated that this combination should be rejected at the 5% confidence level. Notably, the χ² test also failed when we attempted to combine samples a and c. A consistent result was obtained, however, by pooling sample b with sample a and sample c, one at a time. We therefore suggest that the black layer originated from multiple but temporally close fire events, with at least two separate occurrences at 4841 cal BP and 4710 cal BP, approximately 100 years apart. In partial agreement with our hypothesis, the vegetation affected by the fire was not a dense forest, but rather a plant community dominated by tall, likely perennial grass and few scattered trees, a vegetation particularly prone to fire. This suggests that around 4,800 years BP the vegetation of Cervati was more similar to a tall grassland or perhaps a wooded grassland. This event precedes by a few centuries the well-known 4200 BP event (Bini et al., 2019) which marked one of the most significant climate changes of Holocene. Starting from 4200 BP the climate in many regions of the globe has significantly dried out and in some areas even cooled. The event was of such magnitude that several researchers have hypothesized that it contributed to the decline or collapse of several ancient kingdoms (e.g., the Old Kingdom in Egypt, the Akkadian Empire in Mesopotamia, the Liangzhu culture in China) (Weiss, 2016). However, a recent analysis has downplayed the significance and impact of the 4200 BP event, also indicating that a particularly warm period during the Holocene occurred at 4800 BP (McKay et al., 2024), which coincides with the period in which the fires on the Cervati occurred. In our context, a plausible reconstruction is that a particularly warm period fostered the development of vegetation at high elevation, including a high herbaceous biomass that would create the conditions for wildfires. Such fire events, whose origin-natural or anthropogenic-remains uncertain, were likely extensive as it left significant traces in the soil profile. Subsequently, it is likely that the soil was eroded from the slopes of the sinkhole, creating a rocky environment visible today, and leading to the deposition of approximately 1.5 m of soil above the charcoal layer at the sinkhole’s center.

The charcoal, buried in the soil for 4800 years, then underwent a very advanced ageing process. This was clarified by the SEM-EDS analysis which highlighted the accumulation on the surfaces of Al_2_O_3_, known to be present in paleosol as it is not leachable (Sheldon and Tabor, 2009), unlike other cations such as Ca, Na and K. A recent study conducted on charcoal present in kilns in southern Italy forests over carbonate substrates similar to Cervati has shown that much more recent charcoal (aged about 100–200 years) show localized accumulations of Ca, Na and K (Iacomino et al., 2024).

Our study, however, cannot shed light on what happened between the end of the last glaciation and the initial phases of the Holocene. In fact, some evidence shows that during the last glacial maximum even on Mount Cervati there were some small glaciers (Palmentola et al., 1990). In the initial phase of the Holocene the climate was rather warm and humid along the Apennine chain (Giraudi et al., 2011), a factor that could have allowed the development of tree vegetation even at high elevations. To address these questions our research group is carrying out further investigations on Mount Cervati based on molecular methods of ancient DNA (Edwards, 2020) combined with pedo-anthracological analysis of soil layers older than the black layer investigated in this study.

## 5 Conclusions

At Monte Cervati, the remains of an ancient fire buried in a sinkhole were discovered, which we now know temporally preceded the great drought known as the 4200 BP event. This series of such fire events, likely favored by the warm and arid climate, changed the vegetation of the mountain contributing to giving it the appearance with rocky soil and dry grasslands, resembling the current appearance of the summit area of hundreds of mountains in the southern Apennines. Our study shows that in climates with summer drought, intense fire events can cause critical transition with consequent catastrophic shifts in ecosystems that enter an alternate stable state, i.e. woodland or dry grassland. Soil erosion following fire events makes summit areas no longer recolonizable by tree even in historical times. The current state is probably also maintained by the intense grazing still present in summit areas of most of the mountains in Appennines. The study, therefore, indicates that in mountain areas with arid climates in the summer months, global warming is unlikely to cause treeline advancement until other limiting factors like drought and grazing are relaxed. Future research will extend the combined approach of pedo-anthracology with the help of ancient DNA to other mountain groups of the Apennines such as Majella, Velino-Sirente, Sibillini and Pollino to better understand the vegetation history of these areas that experienced antropogenic pressure for millennia.

## Data Availability

The original contributions presented in the study are included in the article/supplementary material. Further inquiries can be directed to the corresponding author.
